# Association between knee osteoarthritis and volumetric bone mineral density

**DOI:** 10.1016/j.ocarto.2025.100740

**Published:** 2026-01-02

**Authors:** Duy K. Hoang, An T. Truong, Linh N. Luu, Tan D. Nguyen, Nhan M. Le, Huy G. Nguyen, Lan T. Ho-Pham, David J. Hunter, Tuan V. Nguyen

**Affiliations:** a University of Technology Sydney, Sydney, Australia; b Saigon Precision Medicine Research Center, Viet Nam; c School of Population Health, UNSW Sydney, Australia; d Sydney Musculoskeletal Health, Kolling Institute, University of Sydney, Sydney, Australia; e Rheumatology Department, Royal North Shore Hospital, Sydney, Australia

**Keywords:** Knee osteoarthritis, Volumetric bone mineral density (vBMD), Tibial cortical bone, Peripheral quantitative computed tomography (pQCT), Dual-energy X-ray absorptiometry (DXA)

## Abstract

**Objective:**

Although patients with radiographic knee osteoarthritis (OA) have a higher areal bone mineral density (BMD) than non-OA individuals, their fracture risk was not significantly different. This study sought to define the association between radiographic knee OA and volumetric BMD.

**Methods:**

We enrolled 944 men and 1506 women aged 40 years or older, selected randomly from Ho Chi Minh City, Vietnam. Knee radiographs were assessed using the Kellgren and Lawrence scale, with grades two or higher indicating knee osteoarthritis (OA). Areal bone mineral density (aBMD) at the femoral neck and lumbar spine was measured using DXA (Hologic Corp, USA). Trabecular and cortical volumetric bone density (vBMD) at the tibia bone were also measured using pQCT XCT2000 (Stratec, Germany). The difference in aBMD and vBMD between individuals with and without OA was quantified using effect size (ES) and 95 % confidence interval (CI).

**Results:**

The prevalence of radiographic knee OA in this cohort was approximately 31 % (n = 755), which increased with age. Compared to non-OA individuals, those with knee OA had higher femoral neck aBMD (ES = 0.04, 95 % CI: 0.02–0.05; P = 0.0002). However, knee OA patients had lower vBMD at the cortical tibia bone (ES = − 8.15, 95 % CI: −14.52 to −1.8; P = 0.01).

**Conclusion:**

These data suggest that approximately a third of Vietnamese people aged 40 and over might have radiographic knee OA. The tibial cortical vBMD was significantly lower in persons with knee OA.

## 1 Introduction

Osteoarthritis (OA) is a disabling joint disease mainly affecting the knees more than other joints [1]. Knee OA has been reported to emerge at earlier ages with increasing prevalence worldwide, including in Asian communities [1]. Meanwhile, there is limited information about the prevalence of knee OA in the Vietnamese community. In addition, knee OA represents a significant socioeconomic challenge, thus highlighting the need for a comprehensive understanding of factors associated with this condition for better medical practice and patient care [2]. Among these factors, growing evidence indicates a close relationship between bone mineral density (BMD) and knee OA [3]. Indeed, previous research has shown areal BMD (aBMD) increases in knee OA patients. However, the fragility fracture rate remains unchanged [3,4]. Besides, cortical bone density has been found to be the main parameter of bone strength, with only a minimal contribution compared to trabecular bone density [5]. However, it remains unclear which of these two parameters of vBMD might correlate with knee OA.

Assessments of BMD involve two main non-invasive methods for measuring: dual-energy x-ray absorptiometry (DXA) and peripheral quantitative computed tomography (pQCT) [6]. The DXA scan is considered the “gold standard” for measuring BMD. However, this method only provides the two-dimensional structure of bone, which cannot differentiate between cortical and trabecular bone [7]. By contrast, pQCT, the second method, calculates volumetric bone mineral density (vBMD) by using a rotating X-ray source to generate cross-sectional images of bone, which are then utilised to evaluate the mineral content per unit volume of bone tissue. This method overcomes the key limitation of DXA by allowing the assessment of three-dimensional vBMD at both trabecular and cortical sites, thus providing better insights into bone strength compared to DXA [7].

Although knee OA and fragility fracture are collectively becoming a burden health issue in developing countries, the association between knee OA and vBMD has not been well documented. We hypothesise that individuals diagnosed with radiographic knee OA might exhibit lower cortical bone density compared to those without radiographic knee OA. This investigation has two specific aims: firstly, to estimate the prevalence of knee OA in older Vietnamese adults, and secondly, to examine the correlation between radiological knee OA and vBMD assessed through pQCT measurements.

## 2 Methods

### 2.1 Study design

This study was part of the Vietnam Osteoporosis Study project initiated in mid-2015, which involves more than 4000 men and women aged 18 years and older in Ho Chi Minh City (formerly Saigon). The study's rationale, protocol, and procedure have been described previously (13). The study's procedure and protocol were approved by the research and ethics committee of the People's Hospital 115 on August 6, 2015 (Approval Number 297/BV-NCKH). The study was conducted according to the ethical principles of the Declaration of Helsinki, and all participants gave written informed consent.

The inclusion criteria of Vietnam Osteoporosis Study were men and women aged 18 years and older who agreed to participate in the study. However, for the current study, only individuals aged 40 years and older were included because OA of the knee mainly affects people in that age range [8]. The following exclusion criteria were applied: previous knee injuries and individuals who were completely paralysed and unable to give informed consent.

### 2.2 Data collection and measurements

Height and body weight were measured by an electronic portable, wall-mounted stadiometer (Seca Model 769; Seca Corp, CA, USA) without shoes, hats, ornaments, or heavy layers of clothing. Body mass index (BMI) was calculated as weight (kg) divided by the square of height (kg/m^2^).

### 2.3 Measurements of aBMD

We first measured aBMD at the femoral neck and lumbar spine (L2-L4) with a Hologic Horizon densitometer (Hologic Corp., Bedford, MA, USA). A qualified radiologist measured aBMD at the femoral neck (FNBMD) and lumbar spine (LSBMD) with aBMD recorded in gram per cm^2^. The densitometer was standardized with a phantom before each measurement. A qualified radiology technologist did the process. The coefficient of variation of BMD measurements was 1.5 % for the lumbar spine and 1.7 % for the femoral neck. Fat mass and lean mass were also derived from the whole-body scan.

### 2.4 Measurements of vBMD for bone architecture assessment

**Fig. 1:**
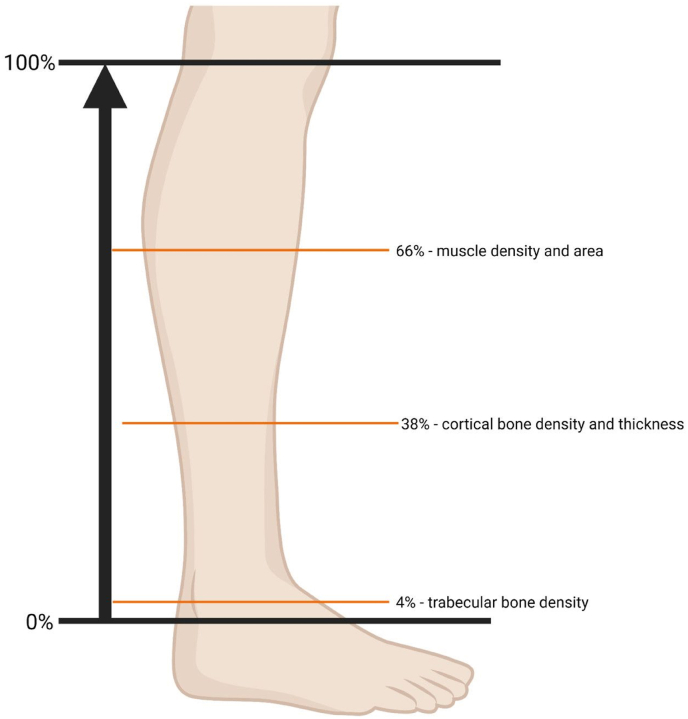
Sites of the scanned leg (for tibia bone).

We used a pQCT XCT2000 (Stratec, Germany) to measure bone volume and bone geometric parameters, including the cortical and trabecular compartments of the lower leg. Three slices were taken in the lower leg at the 4, 38, and 66 % sites (Fig. 1). Cortical vBMD and cortical bone area were evaluated at the 38 % portion of the tibia according to the instructions from the manufacturer [9]. The linear correlation between vBMD at the 38 % site and 66 % site was ∼0.96. Trabecular vBMD and trabecular bone area were evaluated at the 4 % site for the tibia. Based on 20 volunteers, the reproducibility coefficient for the two sites ranged between 1 and 3 %.

### 2.5 Ascertainment of radiographic knee osteoarthritis

The ascertainment of OA was based on a radiographic assessment using the Kellgren - Lawrence scoring system recommended by the WHO as a standard method for studying OA in epidemiologic studies (15). Posterior-anterior (PA) radiographs of both knees were taken from all participants. The radiographs were read by two radiologists who were blinded to each other's readings and were completely unaware of the clinical conditions of the participants. Regarding discrepancy, readings were adjudicated by consensus with a third reader with more than 20 years of experience in rheumatology practice. After image review, the knee with the higher Kellgren–Lawrence (K/L) grade was selected for analysis. The intra- and inter variabilities evaluated for K/L grade (0–4) were confirmed by kappa analysis as sufficient for assessment (0.86 and 0.80, respectively). In each knee, the presence or absence of osteophytes, joint space narrowing, sclerosis and cysts was examined for each knee joint using the Kellgren-Lawrence system of scoring: 0 = none, 1 = possible osteophytes only, 2 = definite osteophytes and possible joint space narrowing, and 4 = large osteophytes, severe joint space narrowing, and/or bony sclerosis. The presence of radiographic OA was defined if the grade was two or more in at least one joint.

### 2.6 Data analysis

The analysis plan was initiated before the data collection and ascertainment of OA of the knee. In the descriptive analysis, we determined the prevalence of radiographic OA in the knee by age group. The primary analysis method was the multiple linear regression model, in which bone parameters were considered dependent variables, and the knee OA group was the independent variable. Four bone measurements employed for analysis include aBMD measured at the lumbar spine and femoral neck, trabecular vBMD, and cortical vBMD at the proximal tibia. The proximal tibia was selected because it is a weight-bearing region near the knee, and previous studies have shown that its trabecular bone density is affected in knee OA, making it a suitable site to detect bone changes [10]. Age and BMI were the covariates in the linear regression model. Before the formal analysis, all outcome variables (e.g., bone parameters) were standardised to have zero mean and unit variance. Effect size showed how many SDs lie between two means and was calculated by dividing the covariance of the independent and dependent variables by the variance of the dependent variable. The test of the null hypothesis with P values being less than 0.05 was considered ‘statistically significant.’ All analyses were done within the R statistical environment.

## 3 Results

The present study included 2450 individuals (1506 women and 944 men) aged 40 years and older (as of 2015), and their demographic and lifestyle characteristics are shown in Table 1 aBMD at lumber spine and femoral neck are lower among women with knee OA compared to non-knee OA. The prevalence of self-reported alcohol consumption and smoking was significantly higher in men than in women (41.6 % vs. 2.61 % and 35.8 % vs. 0.94 %, p < 0.001), respectively.

**Table 1:** Baseline characteristics of the enrolled 2450 participants by knee OA.

Characteristics	Men		Women	
	*Non-knee OA*	*Knee OA*	*Non-knee OA*	*Knee OA*
	*N* = *740*	*N* = *204*	*N* = *955*	*N* = *551*
Age (years)	**54.3 (9.13)**	**59.8 (8.58)**	**53.1 (8.38)**	**61.1 (9.15)**
Weight (kg)	**62.4 (10.0)**	**65.1 (9.25)**	**53.8 (7.49)**	**57.3 (9.39)**
Height (cm)	163 (6.15)	162 (6.81)	**153 (5.39)**	**152 (5.54)**
**Body mass index (kg/m^2^)**	**23.5 (3.22)**	**24.7 (3.11)**	**23.0 (2.87)**	**24.5 (3.28)**
**Lumbar spine BMD (g/cm^2^)**	**0.92 (0.14)**	**0.99 (0.16)**	**0.87 (0.14)**	**0.82 (0.14)**
Femoral neck BMD (g/cm^2^)	0.73 (0.12)	0.76 (0.13)	**0.66 (0.11)**	**0.63 (0.12)**
Lean mass (kg)	42.80 (5.80)	43.10 (5.90)	31.30 (4.30)	31.65 (4.50)
**Fat mass (kg)**	**19.56 (6.0)**	**21.57 (5.73)**	**22.44 (4.80)**	**25.09 (5.20)**
**Percent body fat (%)**	**30.9 (5.47)**	**33.0 (5.72)**	**41.5 (4.54)**	**44.0 (4.19)**
**Overweight + obesity (%)**	**779 (54.4 %)**	**505 (59.2 %)**	**426 (46.7 %)**	**353 (68.0 %)**
**Alcohol consumption (n)**	**322 (44.0 %)**	**66 (32.7 %)**	**32 (3.39 %)**	**7 (1.28 %)**
**Cigarette smoking (n)**	**276 (37.8 %)**	**58 (28.7 %)**	11 (1.17 %)	3 (0.55 %)
**Tibia trab. vBMD (mg/cm^3^)**	202 (31.1)	202 (33.0)	**185 (32.1)**	**175 (33.5)**
**Tibia cort. vBMD (mg/cm^3^)**	1186 (32.1)	1183 (29.8)	**1130 (47.5)**	**1100 (50.1)**

Values are expressed as mean and standard deviation (in bracket).

Bold words indicate significant associations (P < 0.05).

The average age of participants was 55.8 years (SD 9.41), with knee OA patients generally older than non-knee OA individuals. Both women and men with knee OA exhibited significantly higher per cent body fat, leading to a higher prevalence of overweight and obesity than non-OA individuals.

**Fig. 2:**
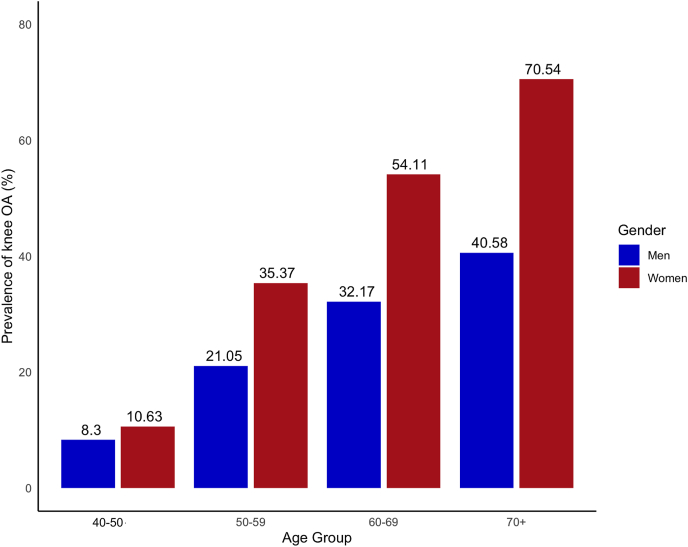
Prevalence of osteoarthritis of the knee classified by age group.

Of the sample, 551 women and 204 men were found to have knee OA based on the Kellgren - Lawrence criteria. Women were more prevalent than men (36.6 % in women vs 21.6 % in men, p < 0.001). The prevalence of radiographic OA of the knee was associated with advancing age (Fig. 2). Among those 50 years of age or younger, the prevalence of knee OA was around 10 % in both genders; this prevalence increased to 50 % in women and 30 % in men between 50 and 59 before rising to 70.54 % among women and 40.58 % in men aged 70 years and older (Fig. 2).

Men with knee OA had higher aBMD than those without knee OA. In men, after adjusting for age and BMI, femoral neck aBMD in OA individuals was higher than those without knee OA by 0.04 standard deviation: ES = 0.04, 95 % CI: 0.02 to 0.05 (P = 0.0002). In women, individuals with knee OA had significantly lower cortical vBMD at the tibia cortical bone than non-knee OA individuals: ES = −8.15, 95 % CI: −14.52 to −1.78 (P = 0.01). (Table 2).

**Table 2:** Association between knee OA and bone parameters after adjusting for age: results of multiple linear regression analysis.

Parameter	Regression coefficient (and standard error) associated with		
	Knee OA	Age	BMI
Men			
Lumbar spine BMD	0.06 (0.01)∗∗	**−0.002 (0.0006)**∗∗	0.01 (0.002)∗∗
Femoral neck BMD	**0.04 (0.01)∗∗**	−0.01 (0.0005)∗∗	0.01 (0.001)∗∗
Tibia trab. bone area	3.80 (7.32)	**−0.21 (0.32)**	0.98 (0.90)
Tibia trab. vBMD	−0.62 (3.12)	**−0.67 (0.14)**∗∗	3.03 (0.38)∗∗
Tibia cort. bone area	5.42 (3.73)	−0.51 (0.17)∗∗	3.42 (0.46)∗∗
Tibia cort. vBMD	3.58 (3.26)	−0.67 (0.14)∗∗	−2.08 (0.40)∗∗
Women			
Lumbar spine BMD	−0.02 (0.01)	−0.01 (0.0005)∗∗	0.01 (0.001)∗∗
Femoral neck BMD	0.0005 (0.01)	−0.01 (0.0004)∗∗	0.01 (0.001)∗∗
Tibia trab. bone area	2.58 (4.27)	0.42 (0.22)	3.07 (0.62)∗∗
Tibia trab. vBMD	−3.36 (2.38)	−1.47 (0.12)∗∗	**3.12 (0.34)∗∗**
Tibia cort. bone area	−0.56 (2.35)	−1.29 (0.12)∗∗	3.27 (0.34)∗∗
Tibia cort. vBMD	**−8.15 (3.25)∗**	−2.91 (0.17)∗∗	0.96 (0.47)∗

Each coefficient represents the number of standard deviations (SD) change in a bone parameter associated with the presence of knee OA, 1-year increase in age and 1 unit of BMI. For example, male patients with knee OA was associated with a 0.04 SD increase in femoral neck BMD, and this association was independent of age and BMI. Significant associations (P < 0.05) are indicated by ∗ and (P < 0.01) are indicated by ∗∗ trab. Trabecular, cort. Cortical.

## 4 Discussion

Knee OA represents a significant public health issue worldwide. However, there needs to be more information available about the prevalence of knee OA in developing countries, including the Vietnamese community. In this study, we identified that the prevalence of radiographic knee OA in a Vietnamese community is as high as in other Asian and Caucasian populations. Additionally, our study observed that Vietnamese women with knee OA had lower cortical vBMD than those without knee OA.

Based on Kellgren-Lawrence criteria, our cohort displayed a radiographic knee OA prevalence of around 31 %, similar to what observed in other Asian countries such as China and Korea [11,12]. Meanwhile, Asian populations such as Thailand and Japan exhibited higher prevalence rates than Vietnam [13,14]. At the continent level, a previous study has revealed that the prevalence of knee OA in Asia surpassed that in North America and Europe [15]. Consistently, among different communities, the prevalence of knee OA has been found to be higher within the Asian community compared to the Caucasian community [16]. This difference might be explained by the cultural practices of bending knees and squatting commonly observed by Asians [17]. Indeed, repetitive knee bending activities, widely recognised as a risk factor for knee OA, increase the probability of damage to the patella cartilage as well as the patellofemoral joint [18,19]. Our study has presented a similar trend of increased knee OA prevalence with advancing age, as observed in other studies [20,21]. Meanwhile, our cohort's lower knee OA prevalence compared to other Asian populations might be attributed to the relatively young age (starting from 40 years) and our participants' generally good health status of our participants.

Based on the threshold KL ≥ 2 for knee OA, our findings revealed a higher prevalence of radiographic knee OA in women than in men. This observation aligns with previous research indicating that women might encounter increased risks of prevalent and incident OA in knees, hips, and hands compared to men [22]. Furthermore, women tend to experience more severe knee OA, especially after menopause, where the drop in their estrogen levels might accelerate knee OA development [22]. The exact cause behind this phenomenon remains unclear; however, it could be linked to reduced cartilage volume and increased cartilage wear [22]. Furthermore, previous literature suggests that bone responses to OA may be compartment- and sex-specific. In postmenopausal women, estrogen deficiency accelerates cortical bone loss [[23], [24]], which may explain the lower cortical vBMD observed in our female OA participants. In contrast, men tend to maintain cortical bone mass for a longer period and may show compensatory increases in aBMD at weight-bearing sites like the femoral neck [22]. This biological divergence may partly account for the sex-specific patterns observed in our results. Additionally, broader differences in mechanical alignment, hormonal variations, genetics, social factors, and other unidentified elements between two genders may also contribute to this condition [25].

Although knee OA has been linked to higher aBMD, individuals with knee OA did not exhibit a reduced fracture risk compared to those without knee OA. This paradoxical relationship has been observed between the Korean [26] and Australian populations [27]. In the Hertfordshire Cohort Study data, Abdin-Mohamed et al. observed that 87 knee OA patients had greater cross-sectional bone area, increasing aBMD [28]. However, it's important to note that aBMD does not accurately represent true bone mineral density, as it only captures a two-dimensional aspect of bone structure [29]. Volumetric BMD, which accounts for the three-dimensional structure of bone, serves as a more reliable measure of bone density.

Our primary finding is a negative significant association between cortical vBMD and knee OA. This observation aligns with another study demonstrating lower vBMD in moderate knee OA [10]. Following previous research, estrogen is of importance for the regulation of trabecular vBMD [30], while cortical vBMD might decrease through several mechanisms: 1) increasing bone resorption from increased parathyroid hormone (PTH) levels [31], reduced weight-bearing exercise, 2) impaired secondary mineralisation of bone, as seen with glucocorticoids [32], and 3) inhibition of osteoblast development resulting in reduced bone formation, influenced by hormones like PTH, and/or lowered GH and IGF-1 levels [33] which is the main component of bone health. The results in our study could be explained by physical activity (which has been shown to be reduced in those with knee OA), which primarily affects the cortical components of bone. First of all, bone size increases with advancing age [34], an adaptation that enhances structural competence, as a larger cross-sectional area and cortical diameter allow for better resistance to bending and compressive loads [35]. Exercise seems to enhance this age-related adaptation, which may improve bone health. Secondly, it is plausible that exercise may counteract natural age-related changes in bone structure [36]. A previous study found increased bone resorption in patients with knee OA [37]. As exercise has been shown to improve both cortical area and cortical BMD and slow bone loss, it may play a role in mediating these effects. Thirdly, studies suggest that patients with knee OA, particularly those with KL grades 3 & 4, experience increased pain and reduced physical activity levels. Taken together, our findings and previous research contribute to why, even though aBMD is increased in people with knee OA, the fracture rate is not lower and shown the important of physical activity on knee OA patients.

Our previous study showed that bone fragility is determined by reduced cortical bone mass in both genders [38]. Therefore, the findings of this study suggest that improving cortical bone density could effectively improve bone health to prevent fractures in knee OA patients. Indeed, a previous study found increased bone resorption in patients with knee OA [37]. Several strategies to reverse this condition would be considered to strengthen the cortical components of the bone, such as increased physical activity and pharmacologic therapy. From the observation in previous study, patients with knee OA, particularly those with KL grades 3 & 4, experience increased pain and reduced physical activity levels [39]. Exercise can increase cortical bone density through various mechanisms. For instance, it promotes bone formation by stimulating osteoblasts for enhanced bone absorption [40]. Weight-bearing exercises, such as walking, running, and resistance training, subject bones to mechanical stress, which signals the body to fortify bones, thereby increasing their density [41]. Additionally, exercise helps reduce the rate of bone loss by slowing down bone resorption by osteoclasts [42]. Regular physical activity also improves bone architecture and distribution, leading to increased vBMD over time.

This study has several strengths and potential limitations. The study was based on a large sample size, and the participants were randomly selected using a rigorous random sampling technique to ensure the representativeness of the general population. The study sample is highly homogeneous, which reduces the impact of potential confounders that could compromise the estimates. Nevertheless, the prevalence estimate from this study was based on radiographs only, not those with concurrent symptoms. In addition, the PA radiographs used in this study primarily visualise the tibiofemoral joint, and the patellofemoral compartment could not be adequately assessed. Therefore, our findings may not fully capture patellofemoral OA. Also, the participants in this study were sampled from an urban population; therefore, the study's findings may not be generalisable to the rural populations. Because participants in this sample were usually healthier volunteers than their counterparts in the general population, the present results could have been biased.

This study's significance lies in managing knee OA patients via bone health improvement. Conventional DXA scans might overlook individuals at high risk, emphasising the necessity of integrating cortical vBMD into bone health for knee OA patients. Knee OA affects millions of older adults worldwide, spanning both developing and developed nations. The association between knee OA and cortical vBMD, a more precise indicator of bone strength compared to aBMD, suggests that improving BMD is crucial for these patients. Achieving this involves strategies such as calcium supplementation, increased physical activity, and physiotherapy, all of which can minimise injury risks and strengthen bones in knee OA patients.

## 5 Conclusion

In summary, based on a population-based study, we observed that 31 % of Vietnam older adults might have knee OA, a prevalence similar to that seen in Caucasian populations. Additionally, we discovered that knee OA patients had lower cortical vBMD in the tibia bone among both genders. These findings highlight the burden of knee OA in Vietnam's aging population and underscore the importance of targeted prevention and management strategies. Future studies should explore the underlying mechanisms and potential interventions to reduce the impact of knee OA on bone health and quality of life.

## Author contributions

DKH, LTHP, and TVN initiated the conception and methods of the study.

DKH, LTHP, and TVN wrote the study protocol.

DKH, LNL, ATT, and NML recruited participants and collected the data in investigating centres.

DKH, LNL, ATT, and HPTL conducted the centralised reading of radiographs.

NML, DKH, TDN, and HGN conducted statistical analyses.

DKH, DJH, and TVN wrote the draft manuscript and initial revision.

## Conflict of interest

DJH is the editor of the osteoarthritis section for UpToDate, co-Editor in Chief of Osteoarthritis and Cartilage and board member of Osteoarthritis Research Society International.

DJH provides consulting advice on scientific advisory boards for Lilly, Sanofi, Novartis, Haleon and Tissuegene.

All other authors have declared no conflicts of interest.
